# Association of Vascular Risk With Severe vs Non-Severe Stroke: An Analysis of the INTERSTROKE Study

**DOI:** 10.1212/WNL.0000000000210087

**Published:** 2024-11-13

**Authors:** Catriona Reddin, Michelle Canavan, Graeme J. Hankey, Shahram Oveisgharan, Peter Langhorne, Xingyu Wang, Helle Klingenberg Iversen, Fernando Lanas, Fawaz Al-Hussain, Anna Czlonkowska, Aytekin Oğuz, Conor Judge, Annika Rosengren, Denis Xavier, Salim Yusuf, Martin J. O'Donnell, for the INTERSTROKE investigators

**Affiliations:** From the HRB Clinical Research Facility Galway (C.R., M.C., C.J., M.J.O.), School of Medicine, University of Galway; Wellcome Trust–HRB (C.R.), Irish Clinical Academic Training, Dublin, Ireland; Institute of Health Informatics (C.R.), University College London, United Kingdom; Perron Institute Chair in Stroke Research (G.J.H.), Medical School, The University of Western Australia; Perron Institute for Neurological and Translational Science (G.J.H.), Perth, Australia; Rush Alzheimer Disease Research Center (S.O.), Rush University Medical Center, Chicago, IL; Academic Section of Geriatric Medicine (P.L.), Glasgow Royal Infirmary, University of Glasgow, United Kingdom; Beijing Hypertension League Institute (X.W.), China; Health and Medical Sciences (H.K.I.), University of Copenhagen, Denmark; Faculty of Medicine (F.L.), Universidad de La Frontera, Temuco, Chile; King Saud University (F.A.-H.), Riyadh, Saudi Arabia; Institute of Psychiatry and Neurology (A.C.), Warsaw, Poland; Department of Internal Medicine (A.O.), Faculty of Medicine, Istanbul Medeniyet University, Turkey; Sahlgrenska University Hospital and Sahlgrenska Academy (A.R.), University of Gothenburg, Sweden; St Johns Medical College and Research Institute (D.X.), Bangalore, India; and Population Health Research Institute (S.Y., M.J.O.), Hamilton Health Sciences and McMaster University, Ontario, Canada.

## Abstract

**Background and Objectives:**

Acute stroke is associated with a spectrum of functional deficits. The objective of this analysis was to explore whether the importance of individual risk factors differ by stroke severity, which may be of relevance to public health strategies to reduce disability.

**Methods:**

INTERSTROKE is an international case-control study of risk factors of first acute stroke (recruitment 2007–August 2015) in 32 countries. Stroke severity was measured using the modified Rankin Scale (mRS) score within 72 hours of admission to hospital. Severe stroke is defined as mRS scores of 4–6 (and non-severe stroke, score of 0–3). We used multinomial logistic regression to estimate comparative odds ratios (ORs; 95% CIs) for severe and non-severe stroke and tested for heterogeneity (*p*_heterogeneity_). We also conducted a matched case-case analysis (matched for age, sex, country, and primary stroke subtype) to determine whether the prevalence of risk factors differed significantly between severe and non-severe stroke. A significant difference in the association of a risk factor of severe stroke compared with non-severe stroke was defined as *p* < 0.05 for both *p*_heterogeneity_ and *p*_case-case_.

**Results:**

Of patients with acute stroke (n = 13,460), 64.0% (n = 8,612) were reported to have mRS scores of 0–3 and 36.0% (n = 4,848) scores of 4–6. The mean age was 61.7 years for patients with non-severe stroke and 62.9 years for patients with severe stroke (*p* = 0.72). 38.1% (n = 3,278) of patients with non-severe stroke and 44.6% (n = 2,162) of patients with severe stroke were female. Hypertension (OR 3.21; 95% CI 2.97–3.47 for severe stroke, OR 2.87; 95% CI 2.69–3.05 for non-severe stroke; *p*_heterogeneity_ = 0.03; *p*_case-case_ < 0.001), atrial fibrillation (OR 4.70; 95% CI 4.05–5.45 for severe stroke, OR 3.61; 95% CI 3.16–4.13 for non-severe stroke; *p*_heterogeneity_ = 0.009; *p*_case-case_ < 0.001), and smoking (OR 1.87; 95% CI 1.72–2.03 for severe stroke, OR 1.65; 95% CI 1.54–1.77 for non-severe stroke; *p*_heterogeneity_ = 0.02; *p*_case-case_ < 0.001) had a stronger association with severe stroke, compared with non-severe stroke. The waist-to-hip ratio had a stronger association with non-severe stroke compared with severe stroke (*p*_heterogeneity_ < 0.001; *p*_case-case_ < 0.001).

**Discussion:**

Hypertension, atrial fibrillation, and smoking had a stronger magnitude of association with severe stroke (compared with non-severe stroke) while the increased waist-to-hip ratio had a stronger magnitude of association with non-severe stroke.

## Introduction

Stroke is a leading preventable cause of disability and death, which is associated with a wide spectrum of disability and dependence.^1^ Acute stroke is associated with a variety of functional deficits and ultimate outcomes, from full recovery to severe dependence and death.^2,3^ Public health interventions to reduce the burden of stroke may reasonably prioritize the prevention of disabling stroke. Accordingly, a detailed knowledge of whether the magnitude of association of traditional vascular risk factors with acute stroke differs by stroke severity may refine global stroke prevention strategies.

A differing association of risk factors with stroke severity may be mediated through a differential association with ischemic stroke vs intracerebral hemorrhage (ICH) because ICH is associated with more severe stroke.^4^ As such, more prominent risk factors of ICH, compared with ischemic stroke, may be expected to translate into more severe stroke syndromes. Within stroke subtypes, there is evidence that some risk factors, particularly atrial fibrillation, may be associated with more severe stroke, but this association is confounded by age, where patients with atrial fibrillation and acute stroke are a decade older than patients without atrial fibrillation.^5,6^ Because the effect of age is controlled in INTERSTROKE, it facilitates assessing the independent association of risk factors among stroke severity syndromes independent of age.

The INTERSTROKE study reported that 10 potentially modifiable risk factors were associated with 90% of the population attributable risk, after controlling for age and sex. In this analysis, we report on whether the magnitude of risk (i.e., odds ratio [OR]) of known risk factors differs by stroke severity among all stroke and by stroke type.

## Methods

INTERSTROKE is an international case-control study of risk factors of first acute stroke. The methods have been described previously.^7^ The aim of the INTERSTROKE study was to establish the association of known and emerging risk factors with stroke and assess the contribution of these risk factors to the burden of stroke. Patients were recruited between January 2007 and August 2015 from 142 centers in 32 countries. Cases were patients who presented with first acute stroke, either ischemic or hemorrhagic, enrolled within 72 hours of hospital admission or 5 days of symptom onset. Cases of ischemic stroke were classified according to the Oxfordshire Community Stroke Project classification as (1) total anterior circulation infarct, (2) partial anterior circulation infarct, (3) posterior circulation infarct, or (4) lacunar infarct, defined by the local physician. Each case was matched for sex and age (±5 years) with a control from the same center/catchment area. Control participants were either community-based or hospital-based. Hospital-based controls were patients admitted to a hospital or those attending an outpatient clinic for disorders or procedures not related to stroke or visitors or relatives of other inpatients. For this analysis, we included cases where the modified Rankin Scale (mRS) score was available and all controls.

## Measurement of Stroke Severity

The mRS score was used to measure stroke severity and measured within 72 hours of admission to hospital. A physician recorded each participant's mRS score at acute presentation. The mRS is a 7-point scale graded as follows: 0—no symptoms; 1—no significant disability despite symptoms; 2—slight disability, unable to perform all previous activities, but independent; 3—moderate disability, requiring some help for bodily needs and/or unable to walk without assistance of a physical device; 4—severe disability, unable to attend to bodily needs without assistance and/or unable to walk without assistance; 5—very severe disability, requiring constant nursing care; 6—death (eMethods).

## Measurement of Risk Factors

Standardized questionnaires were used to collect data on baseline demographics and lifestyle stroke risk factors. Hypertension was defined as a composite of self-reported hypertension and a blood pressure reading of greater than 140/90 mm Hg at recruitment. Atrial fibrillation was defined as a composite of self-reported, documented history or detection by baseline electrocardiogram or during hospitalization (for cases). Diabetes mellitus was defined as the self-reported history of diabetes or hemoglobin A1c ≥6.5%. We measured psychosocial stress in the preceding year using a standardized questionnaire with questions relating to stress at home or work stress. A summary measure of general stress at home and/or in the workplace was generated.^8,9^ We measured depressive symptoms by asking whether, during the past 12 months, the participant had felt sad, blue, or depressed for 2 or more consecutive weeks. Diet quality was measured using a modified Alternative Healthy Eating Index (mAHEI); a higher score indicates a healthier cardiovascular diet. Physical measurements (weight, height, waist-to-hip ratio [WHR]) were recorded in a standardized manner. High WHR was defined as >0.85 in women and >0.9 in men.^10^ Concentrations of ApoB and ApoA1 were measured using nonfasting blood samples, which were collected within 48 hours of recruitment, frozen at −20° to −70°, and shipped to core laboratories for analysis (Hamilton-Canada, Beijing-China, Bangalore-India, and Istanbul-Turkey).

### Statistical Analysis

A dichotomous variable for stroke severity was derived, defining non-severe stroke severity as mRS scores of 0–3 and severe stroke severity as mRS scores of 4–6. The rationale for defining severe stroke as an mRS score of greater than or equal to 4 was to reduce misclassification bias and select true severe stroke phenotypes delineated by independent mobility as a key feature (i.e., patients with independent mobility were classified as those with non-severe stroke).^11^ This approach is further supported by an analysis of health-weighted utility scores associated with the mRS, which showed a statistically significant difference in variance between mRS scores of 0–1 vs 2–5 and 0–2 vs 3–5, but not for 0–3 vs 4–5. These findings suggest that mRS scores of 0–3 and 4–6 represent distinct clinical phenotypes, providing additional justification for dichotomizing stroke severity at this threshold.^12^ In sensitivity analyses, we derived an additional dichotomous variable (alternative dichotomous mRS score), defining non-severe stroke severity as mRS scores between 0 and 2 and severe stroke severity as mRS scores between 3 and 6.

Simple associations were assessed with frequency tables and Pearson χ^2^ tests for 2 independent proportions. Two complementary approaches were used to determine whether the magnitude of association of individual risk factors differed for severe stroke, compared with non-severe stroke. First, we used unconditional multinomial regression analysis to estimate ORs (95% CI) for each risk factor between severe and non-severe stroke, using the entire control group and reporting ORs for severe and non-severe stroke. In these analyses, we tested for heterogeneity between severe and non-severe stroke estimates by dividing the difference in log odds by its standard error.^13^ A *p*_heterogeneity_ of <0.05 was considered statistically significant. Second, we conducted an analysis confined to cases where we compared the prevalence of risk factors between cases with severe stroke and non-severe stroke. In the analysis confined to cases, we matched cases with severe stroke to cases with non-severe stroke by age (±5 years or ±10 years if ≥90 years), sex, country, and stroke type (ischemic or ICH) with patients with non-severe stroke, and conducted a conditional logistic regression multivariable model. A key rationale for the latter analysis was to minimize a residual confounding effect of differences in risk factor prevalence by country and region. Therefore, we used 2 complementary analyses to determine whether the association of common risk factors differed by stroke severity. Exposures included in the analysis were hypertension, atrial fibrillation, diabetes mellitus, smoking, alcohol use, diet quality (AHEI tertile), high WHR, physical inactivity, psychosocial stress, ApoB, and ApoA1. All models were adjusted for age and mutually adjusted for other risk factor exposures (with the exception of ApoB and ApoA1 due to missing data for these variables). We considered there to be a significant difference in the association of a risk factor with severe stroke compared with non-severe stroke if both analyses met prespecified statistical significance (*p* < 0.05).

Sensitivity analyses were performed using an alternative dichotomized mRS score (0–2 vs 3–6) because this is used commonly in randomized controlled trials.^14^ A complete case analysis was the approach used to address missing data (n = 2 missing observations for the primary outcome measure). Statistical analyses were performed using R version 4.3.2.

### Standard Protocol Approvals, Registrations, and Patient Consents

The INTERSTROKE study was approved by the ethics committees in all participating centers. Written informed consent was obtained from participants or their proxy. This study adheres to Strengthening the Reporting of Observational Studies in Epidemiology reporting guidelines.

### Data Availability

Information on the design and rationale of INTERSTROKE has been previously published. Individual participant data will not be made available.

## Results

A total of 13,462 patients with stroke (cases) and 13,488 matched controls were recruited between January 2007 and August 2015. For this analysis, we included 13,460 cases where the mRS score was available and 13,488 controls. Of participants (cases) with acute stroke (n = 13,460), 64.0% (n = 8,612) were reported to have mRS scores of 0–3 and 36.0% (n = 4,848) mRS scores of 4–6. The characteristics of the participants are reported by stroke severity and country income level, which reported older age among patients presenting with severe stroke (Table 1 and eTable 1). The mean age was 61.7 years for patients with non-severe stroke and 62.9 years for patients with severe stroke (*p* = 0.72). However, when stratified by country income level, patients with non-severe stroke were younger than patients with severe stroke (eTable 1). The highest proportion of patients with severe stroke was in South Asia (57.3% [n = 1,642]) and Africa (53.9% [n = 525]), with the lowest proportion of patients with severe stroke in Western Europe/North America/Australasia (18.2% [n = 348]) (Figure). The characteristics of matched case-case cohort (patients with severe stroke, mRS scores 4–6, matched with patients with non-severe stroke, mRS scores 0–3) are reported in eTable 2. ICH stroke type was more common among patients with severe stroke than in patients with non-severe stroke (35.7% vs 15.4%) (Figure and eTable 3). Among ischemic stroke cases, three-quarters (356/477) of participants with a total anterior circulation syndrome had a severe stroke (Figure and eTable 3).

**Table 1:** Patient Characteristics of Controls and Cases (by Stroke Severity [mRS Scores 0–3 vs 4–6])

Variable	Overall (N = 26,948)^a^	Control (N = 13,488)^a^	Non-severe stroke (N = 8,612)^a^	Severe stroke (N = 4,848)^a^	*p* Value^b^
Age, y	61.74 (13.44)	61.31 (13.29)	61.71 (13.35)	62.98 (13.94)	<0.001
Sex					<0.001
Female	10,893 (40.42)	5,453 (40.43)	3,278 (38.06)	2,162 (44.60)	
Male	16,055 (59.58)	8,035 (59.57)	5,334 (61.94)	2,686 (55.40)	
Region					<0.001
W. Europe/North America/Australasia	3,836 (14.23)	1,919 (14.23)	1,569 (18.22)	348 (7.18)	
Eastern/Central Europe/Middle East	2,787 (10.34)	1,393 (10.33)	1,027 (11.93)	367 (7.57)	
Africa	1,949 (7.23)	976 (7.24)	448 (5.20)	525 (10.83)	
South Asia	5,734 (21.28)	2,870 (21.28)	1,222 (14.19)	1,642 (33.87)	
China	7,973 (29.59)	3,987 (29.56)	2,779 (32.27)	1,207 (24.90)	
South East Asia	1,710 (6.35)	855 (6.34)	626 (7.27)	229 (4.72)	
South America	2,959 (10.98)	1,488 (11.03)	941 (10.93)	530 (10.93)	
Education					<0.001
None	3,791 (14.07)	1,635 (12.13)	1,040 (12.08)	1,116 (23.02)	
1–12 y	16,259 (60.35)	7,787 (57.75)	5,584 (64.85)	2,888 (59.57)	
Trade school or university	6,892 (25.58)	4,062 (30.12)	1,986 (23.07)	844 (17.41)	
Unknown	6	4	2	0	
History of hypertension or adjusted BP >140/90 at admission	16,159 (59.96)	6,385 (47.34)	6,183 (71.80)	3,591 (74.07)	<0.001
History of atrial fibrillation/flutter	1,699 (6.30)	370 (2.74)	783 (9.09)	546 (11.26)	<0.001
Smoking history					<0.001
Never or former smoker	19,840 (73.66)	10,464 (77.62)	6,000 (69.71)	3,376 (69.67)	
Current smoker	7,094 (26.34)	3,017 (22.38)	2,607 (30.29)	1,470 (30.33)	
Unknown	14	7	5	2	
Leisure physical activity					<0.001
Mainly inactive	23,337 (86.66)	11,279 (83.68)	7,581 (88.08)	4,477 (92.42)	
Mainly active	3,593 (13.34)	2,200 (16.32)	1,026 (11.92)	367 (7.58)	
Unknown	18	9	5	4	
Alcohol use					<0.001
Never or former	20,481 (76.02)	10,459 (77.55)	6,184 (71.83)	3,838 (79.18)	
Current	6,461 (23.98)	3,027 (22.45)	2,425 (28.17)	1,009 (20.82)	
Unknown	6	2	3	1	
Total AHEI score	23.05 (6.44)	23.48 (6.58)	22.80 (6.38)	22.31 (6.03)	<0.001
Body mass index (kg/m^2^)	25.71 (4.81)	25.59 (4.74)	26.12 (4.74)	25.28 (5.10)	<0.001
Unknown	340	91	58	191	
Waist-to-hip ratio	0.93 (0.08)	0.92 (0.08)	0.94 (0.08)	0.93 (0.08)	<0.001
Unknown	249	39	97	113	
History of diabetes or HbA1c ≥6.5%	6,733 (25.00)	2,957 (21.94)	2,348 (27.27)	1,428 (29.48)	<0.001
Unknown	17	10	3	4	
ApoB	0.98 (0.28)	0.97 (0.27)	0.99 (0.29)	0.97 (0.30)	<0.001
Unknown	2,793	1,425	639	729	
ApoA1	1.32 (0.33)	1.38 (0.34)	1.29 (0.31)	1.24 (0.33)	<0.001
Unknown	2,922	1,521	648	753	
Depression					<0.001
Not sad in past 2 wk	22,502 (83.76)	11,579 (85.87)	6,989 (81.41)	3,934 (82.06)	
Sad in past 2 wk	4,362 (16.24)	1,906 (14.13)	1,596 (18.59)	860 (17.94)	
Unknown	84	3	27	54	
Global stress					<0.001
None or some periods	22,133 (82.56)	11,529 (85.64)	6,779 (79.21)	3,825 (79.85)	
Several periods or permanent	4,677 (17.44)	1,933 (14.36)	1,779 (20.79)	965 (20.15)	
Unknown	138	26	54	58	
Modified Rankin Scale score before stroke					
0	11,763 (87.41)	0 (NA)	7,711 (89.55)	4,052 (83.60)	
1	1,113 (8.27)	0 (NA)	636 (7.39)	477 (9.84)	
2	374 (2.78)	0 (NA)	185 (2.15)	189 (3.90)	
3	170 (1.26)	0 (NA)	72 (0.84)	98 (2.02)	
4–5	38 (0.28)	0 (NA)	7 (0.08)	31 (0.64)	
NA	13,488	13,488	—	—	
Unknown	2		1	1	
Current modified Rankin Scale score					
0	451 (3.35)	—	451 (5.24)	—	
1	2,120 (15.75)	—	2,120 (24.62)	—	
2	2,651 (19.70)	—	2,651 (30.78)	—	
3	3,390 (25.19)	—	3,390 (39.36)	—	
4	3,009 (22.36)	—	—	3,009 (62.07)	
5–6	1,839 (13.66)	—	—	1,839 (37.93)	
NA	13,488	13,488	—	0	
Stroke types					<0.001
Ischemic	10,361 (38.45)	—	7,261 (84.31)	3,100 (63.94)	
ICH	3,055 (11.34)	—	1,324 (15.37)	1,731 (35.71)	
NA	13,532 (50.22)	13,488 (100.00)	27 (0.31)	17 (0.35)	
Final OCSP classification					
TACI	661 (6.38)	—	271 (3.73)	390 (12.59)	
PACI	4,818 (46.51)	—	3,313 (45.63)	1,505 (48.58)	
POCI	1,489 (14.38)	—	1,121 (15.44)	368 (11.88)	
LACI	2,743 (26.48)	—	2,133 (29.38)	610 (19.69)	
Other	647 (6.25)	—	422 (5.81)	225 (7.26)	
NA	16,590	13,488	1,352	1,750	
TOAST classification					
TOAST cardioembolic	1,198 (11.52)	—	800 (10.98)	398 (12.79)	
TOAST large vessel	2,118 (20.37)	—	1,186 (16.28)	932 (29.94)	
TOAST small vessel	4,007 (38.53)	—	3,082 (42.29)	925 (29.71)	
TOAST undetermined	2,307 (22.18)	—	1,729 (23.73)	578 (18.57)	
Other TOAST classification	770 (7.40)	—	490 (6.72)	280 (8.99)	
Unknown	16,548	13,488	1,325	1,735	

Abbreviations: AHEI = Alternative Healthy Eating Index; BP = blood pressure; HbA1C = hemoglobin A1c; ICH = intracerebral hemorrhage; LACI = lacunar circulation infarct; mRS = modified Rankin Scale; NA = not available; OCSP = Oxfordshire Community Stroke Project; PACI = partial anterior circulation infarct; POCI = posterior circulation infarct; TACI = total anterior circulation infarct; TOAST = Trial of ORG 10172 in Acute Stroke Treatment.

Missing mRS: n = 2.

a Mean (SD); n (%).

b Kruskal-Wallis rank-sum test; Pearson χ^2^ test.

**Figure:**
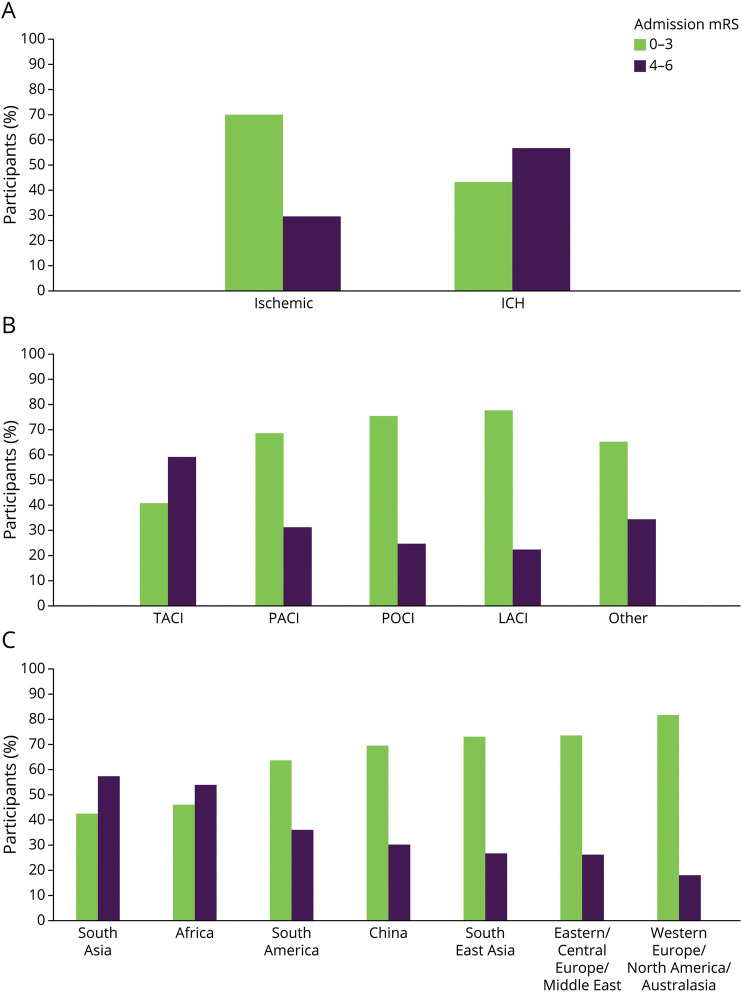
Stroke Severity by Stroke Type and Region Bar chart depicting stroke severity by stroke type, OCSP, and region, respectively. The x-axis represents stroke type (panel A), OCSP classification (panel B), and region (panel C), and the y-axis represents the percentage of case participants. The green bar represents non-severe stroke severity (mRS scores 0–3) and the purple bar severe stroke severity (mRS scores 4–6). Bar chart depicting OCSP classification only includes ischemic stroke cases. LACI = lacunar circulation infarct; mRS = modified Rankin Scale; OCSP = Oxfordshire Community Stroke Project; PACI = partial anterior circulation infarct; POCI = posterior circulation infarct; TACI = total anterior circulation infarct.

Three risk factors were associated with significantly higher odds of severe stroke (vs non-severe stroke), namely hypertension, atrial fibrillation, and smoking. Hypertension was associated with increased odds of both severe stroke and non-severe stroke for all stroke, with a statistically significant difference between estimates and higher odds of severe stroke compared with non-severe stroke among matched cases (severe stroke OR 3.21; 95% CI 2.97–3.47; non-severe stroke OR 2.87; 95% CI 2.69–3.05; *p*_heterogeneity_ = 0.03; *p*_case-case_ < 0.001) (Table 2). This was consistent for ischemic stroke (*p*_case-case_ = 0.002) but not ICH (*p*_case-case_ = 0.17) (eTable 4). Atrial fibrillation was associated with increased odds of both severe stroke and non-severe stroke for all stroke, with a statistically significant difference between estimates and higher odds of severe stroke compared with non-severe stroke among matched cases (severe stroke OR 4.70; 95% CI 4.05–5.45; non-severe stroke OR 3.61; 95% CI 3.16–4.13; *p*_heterogeneity_ = 0.009; *p*_case-case_ < 0.001) (Table 2). This was consistent for ischemic stroke (*p*_case-case_ < 0.001) but not ICH (*p*_case-case_ = 0.44) (eTable 4). Smoking was associated with increased odds of both severe stroke and non-severe stroke for all stroke, with a statistically significant difference between estimates and higher odds of severe stroke compared with non-severe stroke among matched cases (severe stroke OR 1.87; 95% CI 1.72–2.03; non-severe stroke OR 1.65; 95% CI 1.54–1.77; *p*_heterogeneity_ = 0.02; *p*_case-case_ < 0.001) (Table 2). This was consistent for ischemic stroke (*p*_case-case_ < 0.001) and ICH (*p*_case-case_ 0.02) (eTable 4).

**Table 2:** Association of Risk Factors With Stroke by Stroke Severity

Risk factor	Multinomial logistic regression All stroke			Conditional logistic regression All stroke
	Outcome	Odds ratio (95% CI)^a^	*p* Value for heterogeneity	*p* Value case-case
Hypertension	Severe stroke	3.21 (2.97–3.47)	0.03	<0.001
	Non-severe stroke	2.87 (2.69–3.05)		
Atrial fibrillation	Severe stroke	4.70 (4.05–5.45)	0.009	<0.001
	Non-severe stroke	3.61 (3.16–4.13)		
Smoking: current smoker	Severe stroke	1.87 (1.72–2.03)	0.02	<0.001
	Non-severe stroke	1.65 (1.54–1.77)		
High WHR	Severe stroke	1.11 (1.02–1.20)	<0.001	<0.001
	Non-severe stroke	1.37 (1.28–1.47)		
Physical activity (mainly inactive)	Severe stroke	2.05 (1.81–2.32)	<0.001	0.81
	Non-severe stroke	1.47 (1.34–1.60)		
Alcohol use (current)	Severe stroke	0.99 (0.90–1.09)	<0.001	0.06
	Non-severe stroke	1.30 (1.21–1.40)		
Diabetes mellitus	Severe stroke	1.29 (1.19–1.39)	0.06	0.21
	Non-severe stroke	1.17 (1.10–1.26)		
Global stress: several periods/permanent	Severe stroke	1.62 (1.48–1.78)	0.24	0.22
	Non-severe stroke	1.51 (1.40–1.63)		
AHEI tertile: tertile 1	Severe stroke	1.28 (1.19–1.38)	0.25	0.86
	Non-severe stroke	1.21 (1.14–1.29)		
ApoB >1^b^	Severe stroke	0.97 (0.90–1.05)	0.006	0.11
	Non-severe stroke	1.11 (1.05–1.18)		
ApoA1^b^: tertile 1	Severe stroke	2.36 (2.17–2.56)	<0.01	0.25
	Non-severe stroke	1.52 (1.42–1.62)		

Abbreviations: AHEI = Alternative Healthy Eating Index; WHR = waist-to-hip ratio.

Reference levels: smoking, never/former smoker; physical activity, mainly active; global stress, none or some periods; AHEI, tertile 2/3; ApoA1, tertile 2/3.

a Adjusted for age, sex, and country and mutually adjusted for risk factors.

b Owing to missing data, not included in the multivariable model.

Diabetes mellitus, lower diet quality (mAHEI tertile 1), physical inactivity, and global stress were each associated with increased odds of both severe stroke and non-severe stroke for all stroke, with no significant difference in odds of severe stroke compared with non-severe stroke among matched cases (Table 2). Alcohol use was associated with increased odds of non-severe stroke but not severe stroke for all stroke (severe stroke OR 0.99; 95% CI 0.90–1.09; non-severe stroke OR 1.30; 95% CI 1.21–1.40; *p*_heterogeneity_ < 0.001). Alcohol use was associated with no significant difference in odds of severe stroke compared with non-severe stroke among matched cases (*p*_case-case_ < 0.06). High ApoB (>1) was associated with increased odds of non-severe stroke but not severe stroke for all stroke, with no significant difference in odds of severe stroke compared with non-severe stroke among matched cases (severe stroke OR 0.97; 95% CI 0.90–1.05; non-severe stroke OR 1.11; 95% CI 1.05–1.18; *p*_heterogeneity_ = 0.006; *p*_case-case_ = 0.11) (Table 2).

High WHR was associated with increased odds of both severe stroke and non-severe stroke for all stroke, with a statistically significant difference between estimates and lower odds of severe stroke compared with non-severe stroke among matched cases (severe stroke OR 1.11; 95% CI 1.02–1.20; non-severe stroke OR 1.37; 95% CI 1.28–1.47; *p*_heterogeneity_ < 0.001; *p*_case-case_ < 0.001) (Table 2). Low ApoA1 (tertile 1) was associated with increased odds of both severe stroke and non-severe stroke, with a higher magnitude of risk associated with severe compared with non-severe stroke, with no significant difference in odds of severe stroke compared with non-severe stroke among matched cases (severe stroke OR 2.36; 95% CI 2.17–2.56; non-severe stroke OR 1.52; 95% CI 1.42–1.62; *p*_heterogeneity_ < 0.001; *p*_case-case_ = 0.25) (Table 2).

Sensitivity analysis was performed by alternative mRS score dichotomy, which did not materially alter results (eTable 5).

## Discussion

In this large international case-control study of risk factors of stroke, we observed that hypertension, atrial fibrillation, and smoking were associated with higher odds of severe stroke (vs non-severe stroke) while higher WHR was associated with lower odds of severe stroke. However, all risk factors were significantly associated with both severe and non-severe stroke syndromes, but to varying magnitudes.

Hypertension is the most important modifiable risk factor of stroke, globally.^1,7,14,15^ Hypertension is also a stronger risk factor of ICH, compared with ischemic stroke, and because ICH is also associated with greater stroke severity, we had expected a stronger magnitude of association between hypertension and severe stroke.^1,7,15,16^ We did observe a higher odds ratio between hypertension and severe stroke (compared with non-severe stroke), which was observed for both ischemic and ICH stroke types. Therefore, the difference in magnitude of odds ratio is not primarily due to a different magnitude of risk with ICH. Previous studies have reported an association of hypertension with severe stroke. Within ischemic stroke subtype, this may be related to higher prevalence of subclinical atrial fibrillation, as hypertension is a strong risk factor of atrial fibrillation.^17-19^ Our findings emphasize the importance of hypertension control in stroke prevention.^20-23^ This has particular relevance for lower and middle-income countries who have rapidly increasing rates of hypertension and strokes at younger age.^20^

Our study reports that atrial fibrillation is associated with a higher odds ratio of severe stroke compared with non-severe stroke, which is directionally consistent with previous studies.^5,24,25^ However, previous studies have generally reported a more marked difference in magnitude of risk between stroke severity syndromes.^6^ Populations with atrial fibrillation differ from populations without atrial fibrillation, particularly regarding age. In a previous cohort study exploring determinants of the association of atrial fibrillation with severe stroke, the mean age of patients with atrial fibrillation was 10 years older than of patients without atrial fibrillation.^3,6,24^ In our analyses, we controlled for the confounding effect of age and so our estimates reflect an independent effect of atrial fibrillation. The mechanism through which atrial fibrillation increases stroke severity is likely related to the larger size of an embolic clot. This is consistent with our observed higher proportion of patients presenting with total anterior circulation ischemic stroke.

We report that stronger association of current smoking status with severe stroke (vs non-severe stroke) syndromes may relate to a stronger mechanistic relationship with large vessel atherosclerotic disease, compared with other stroke etiologies.^26^ Within ischemic stroke subtypes, a different magnitude of association between vascular risk factors and etiologic subtypes of stroke may translate into differing associations with stroke severity, given that ischemic stroke attributable to large vessel disease and cardioembolism are associated with greater stroke severity, compared with small vessel mechanisms.

Our observed association of high WHR with non-severe stroke is consistent with previous studies that reported a higher magnitude of association with small vessel disease compared with large vessel or cardioembolic etiologies.^27^ Similarly, an analysis of the Telemedical Project for Integrative Stroke Care trial reported lower odds of mRS scores 3–6 in those who were overweight or obese (body mass index 30–35)^28^ while a single-country retrospective study reported that high WHR (using an alternative definition of high WHR of >0.78 and >0.92) was associated with increased odds of severe stroke (mRS scores 3–6).^29^

Our study has some potential limitations, including the possible influence of unmeasured confounders. We used 2 analytic approaches to determining whether the relative importance of risk factors differed by stroke severity, and our conclusions were based on statistical significance for both models. In general, findings from both analyses were consistent, with the exception of physical activity and alcohol intake, which were significant on the multinomial model but not the matched case-case analysis. Alcohol intake varies by region and sex, and significant findings on multinomial regression were likely related to a residual confounding effect from these variables. We would consider our analytic approach and higher threshold for significance to be an advantage of our study. Our study may have sources of unmeasured confounding. For example, smoking is associated with other comorbidities (e.g., chronic obstructive pulmonary disease and cancer) that may contribute to increased stroke severity, especially because we measured stroke severity with the mRS rather than with a stroke severity neurologic scale, such as the NIH Stroke Scale. As such, other comorbidities and frailty may affect the mRS score through non-stroke mechanisms. It is also important to note that the mRS score may underestimate stroke severity in those in whom mobility is unaffected. A case-control design has inherent limitations, such as challenges with including a representative sample of patients across the stroke spectrum, and those with severe stroke are challenging to recruit into research studies, given difficulties in communicating with patients. Despite these limitations, an incident case-control design provides distinct advantages in addressing the current research question, in that it allows real-time standardized measurement of the stroke severity (i.e., shortly after admission).

The relative importance of some vascular risk factors varies by stroke severity, although each risk factor was significantly associated with severe and non-severe stroke. We observed that hypertension, atrial fibrillation, and smoking were associated with higher odds of severe stroke while higher WHR was associated with higher odds of non-severe stroke, compared with severe stroke. Our results support efforts to control hypertension, atrial fibrillation, and smoking to prevent severe, disabling stroke.

## Appendix 1 Authors

**Table:**

Name	Location	Contribution
Catriona Reddin, MB BCh, BAO, MSc	HRB Clinical Research Facility Galway, School of Medicine, University of Galway; Wellcome Trust–HRB, Irish Clinical Academic Training, Dublin, Ireland; Institute of Health Informatics, University College London, United Kingdom	Drafting/revision of the manuscript for content, including medical writing for content; analysis or interpretation of data
Michelle Canavan, PhD	HRB Clinical Research Facility Galway, School of Medicine, University of Galway, Galway, Ireland	Drafting/revision of the manuscript for content, including medical writing for content; analysis or interpretation of data
Graeme J. Hankey, MBBS, MD, FRACP	Perron Institute Chair in Stroke Research, Medical School, The University of Western Australia; Perron Institute for Neurologic and Translational Science, Perth, Australia	Drafting/revision of the manuscript for content, including medical writing for content; major role in the acquisition of data; analysis or interpretation of data
Shahram Oveisgharan, MD	Rush Alzheimer Disease Research Center, Rush University Medical Center, Chicago, IL	Drafting/revision of the manuscript for content, including medical writing for content; major role in the acquisition of data; analysis or interpretation of data
Peter Langhorne, PhD	Academic Section of Geriatric Medicine, Glasgow Royal Infirmary, University of Glasgow, United Kingdom	Drafting/revision of the manuscript for content, including medical writing for content; major role in the acquisition of data; analysis or interpretation of data
Xingyu Wang, PhD	Beijing Hypertension League Institute, China	Drafting/revision of the manuscript for content, including medical writing for content; major role in the acquisition of data; analysis or interpretation of data
Helle Klingenberg Iversen, MDSci	Health and Medical Sciences, University of Copenhagen, Denmark	Drafting/revision of the manuscript for content, including medical writing for content; study concept or design; analysis or interpretation of data
Fernando Lanas, MD, MSc, PhD	Faculty of Medicine, Universidad de La Frontera, Temuco, Chile	Drafting/revision of the manuscript for content, including medical writing for content; study concept or design; analysis or interpretation of data
Fawaz Al-Hussain, MD	King Saud University, Riyadh, Saudi Arabia	Drafting/revision of the manuscript for content, including medical writing for content; major role in the acquisition of data; analysis or interpretation of data
Anna Czlonkowska, MD, PhD	Institute of Psychiatry and Neurology, Warsaw, Poland	Drafting/revision of the manuscript for content, including medical writing for content; major role in the acquisition of data; analysis or interpretation of data
Aytekin Oğuz, MD	Department of Internal Medicine, Faculty of Medicine, Istanbul Medeniyet University, Turkey	Drafting/revision of the manuscript for content, including medical writing for content; major role in the acquisition of data; analysis or interpretation of data
Conor Judge, PhD	HRB Clinical Research Facility Galway, School of Medicine, University of Galway, Galway, Ireland	Drafting/revision of the manuscript for content, including medical writing for content; analysis or interpretation of data
Annika Rosengren, MD	Sahlgrenska University Hospital and Sahlgrenska Academy, University of Gothenburg, Sweden	Drafting/revision of the manuscript for content, including medical writing for content; major role in the acquisition of data; analysis or interpretation of data
Denis Xavier, MD	St Johns Medical College and Research Institute, Bangalore, India	Drafting/revision of the manuscript for content, including medical writing for content; major role in the acquisition of data; analysis or interpretation of data
Salim Yusuf, DPhil	Population Health Research Institute, Hamilton Health Sciences and McMaster University, Ontario, Canada	Drafting/revision of the manuscript for content, including medical writing for content; major role in the acquisition of data; study concept or design; analysis or interpretation of data
Martin J. O'Donnell, PhD	HRB Clinical Research Facility Galway, School of Medicine, University of Galway, Galway, Ireland; Population Health Research Institute, Hamilton Health Sciences and McMaster University, Ontario, Canada	Drafting/revision of the manuscript for content, including medical writing for content; major role in the acquisition of data; study concept or design; analysis or interpretation of data

## Appendix 2 Coinvestigators

**Table:**

Coinvestigators are listed at Neurology.org.

## Glossary

ICH: intracerebral hemorrhage
mAHEI: modified Alternative Healthy Eating Index
mRS: modified Rankin Scale
OR: odds ratio
WHR: waist-to-hip ratio
